# Preventive Effect of Liothyronine on Electroconvulsive Therapy-Induced Memory Deficit in Patients with Major Depressive Disorder: A Double-Blind Controlled Clinical Trial

**DOI:** 10.1155/2015/503918

**Published:** 2015-04-06

**Authors:** Arash Mohagheghi, Asghar Arfaie, Shahrokh Amiri, Masoud Nouri, Salman Abdi, Salman Safikhanlou

**Affiliations:** Research Center of Psychiatry and Behavioral Sciences (RCPBS), Tabriz University of Medical Sciences, Tabriz 51677, Iran

## Abstract

*Introduction and Objective*. Despite the effectiveness of electroconvulsive therapy (ECT) in treating major depressive disorder (MDD), its cognitive side effects make it less popular. This study investigated the impact of liothyronine on ECT-induced memory deficit in patients with MDD. *Methodology*. This is a double-blind clinical trial, in which 60 patients with MDD who were referred for ECT were selected. The diagnosis was based on the criteria of DSM-IV-TR. Patients were divided randomly into two groups to receive either liothyronine (50 mcg every morning) or placebo. After the assessment with Wechsler Memory Scale-Revised (WMS-R) before first session of ECT, posttests were repeated again, two months after the completion of ECT. *Findings*. By controlling the pretest scores, the mean scores of the experimental group were higher than the control group in delayed recall, verbal memory, visual memory, general memory, and attention/concentration scales (*P* < 0.05). *Conclusion*. Liothyronine may prevent ECT-induced memory impairment in patients with MDD. This study has been registered in IRCT under IRCT201401122660N2.

## 1. Introduction 

Convulsive interventions have been used to treat mental disorders since the 16th century up to the moment in the form of electroconvulsive therapy (ECT) [1]. The most common therapeutic indication of ECT is major depression disorder (MDD) and its effectiveness in reducing depressive symptoms has been confirmed in several studies [2]. However, the cognitive complications of ECT have been reported as the main limitation for its use. These side effects occur more in patients with depression. Cognitive side effects and memory deficits are considered as a major limitation to the use of ECT, with 12.4% prevalence for permanent anterograde amnesia in a community setting [3, 4]. Despite this, almost all patients return to their previous cognitive status within six months. However, some patients seriously complain of permanent drawbacks in their memory [3].

Disorientation, destruction of processing speed, anterograde and retrograde amnesia, impaired visual and spatial function, and word finding difficulty usually occur immediately after an ECT session. Except for anterograde memory impairment, other cognitive effects of ECT return to the baseline. Anterograde memory improvement occurs gradually, but point defects may still remain. Fraser et al. [5] have also shown that memory may be affected by ECT for a short-term (less than six months).

Falconer et al. [6] showed that memory problems can be resolved after a one-month intervention. This study used Cambridge Neuropsychological Test Automated Battery (CANTAB) for cognitive assessments and showed that impairment in spatial recognition may be observed two weeks after ECT. Another study reported short-term and long-term deficits in autobiographical memory occurring shortly and two months after ECT, respectively, in patients with MDD. These patients had a poor performance in stating the exact details of stories and most had difficulty in remembering stories of others compared with their personal biography. Retrograde amnesia was reduced after 2 months of follow-up, but impairment in remembering the recent public events in detail continued still [7].

Although various medical interventions are suggested to reduce the cognitive deficits after ECT, no specific medicine has been found for improving them. Numerous studies investigated the preventive effects of galantamine [8], physostigmine [9], naloxone [10], dexamethasone [11], and piracetam [12] on ECT-associated memory deficit. Some have shown the positive effect of thyroid hormones on cognitive side effects of ECT [13]. However, there is a high tendency to use N-methyl-D-aspartate antagonists such as anesthetic ketamine and thyroid hormone. There also is some information about the use of physostigmine, thyroid hormone, and naloxone, indicating that they can reduce the psychological effects of ECT, but their effects are not well studied and identified yet. Liothyronine is used to increase the response to ECT and if administered at the beginning of an ECT course, response time and the incidence of cognitive problems are reduced [4]. In a study evaluating effects of liothyronine, piracetam, and placebo, biographical memory and mental control did not decrease in the liothyronine group, but orientation declined gradually until the last session of ECT and then significantly increased in a one-month follow-up [12].

Despite the effectiveness of ECT in treatment of MDD patients have concerns about the cognitive effects of ECT and memory deficits in particular. The main incentives of this research are the increasing use of ECT in treatment of MDD, scientific need for more precise information about it, and suggestion of previous researchers for further complementary studies. Accordingly, the present study investigates the effectiveness of liothyronine on ECT-induced memory deficit in patients with MDD.

## 2. Methodology

The study protocol was approved by the Institutional Review Board of Tabriz University of Medical Sciences in accordance with the principles of the Declaration of Helsinki. This trial is registered with the Iranian Clinical Trials Registry (IRCT: IRCT201401122660N2).

This is a double-blind clinical trial, in which the evaluator and the patients were unaware of the medication received by the patients (liothyronine or placebo). Sixty inpatients and outpatients with MDD who were referred to the ECT Ward, Razi Psychiatric Hospital, Tabriz, Northwest of Iran, were enrolled in this study. The diagnosis was based on the criteria of DSM-IV-TR, using the structured clinical interview for DSM-IV (SCID-IV). The inclusion criteria were the diagnosis of MDD, giving written informed consent, age between 20 to 50 years, Being candidate for receiving ECT based on diagnosis of a psychiatrist, and no history of ECT during the last 6 months. The exclusion criteria were any contraindications of liothyronine, drug and alcohol abuse, organic brain disorders, mental retardation (based on the patients' history, physical examination, and medical records). Patients who experienced ECT-induced delirium or completed less than 6 sessions for any reasons were also excluded.

Selected patients were randomly assigned to experimental and control groups and assessed by the Wechsler Memory Scale-Revised (WMS-R) test as described later. The two groups were matched in terms of depression severity. Then the experimental and control groups received liothyronine and placebo, respectively. Two months after the end of ECT sessions, WMS-R posttest was performed for both groups. For the intervention group, liothyronine (two tablets of 25 mcg) was administered orally from the day before beginning of ECT every morning until the last session. Lactose tablets which were similar to liothyronine in appearance were used as placebo in this study with the same directions as liothyronine for the intervention group. Patients were asked to stop any other medications during the study.

ECT was administered by Thymatron DGX device (Somatics, ILC, lake Bluff, USA) through bilateral technique following a dose titration method. A bilateral dosage at 50–100% above seizure threshold of the patient (the first stimulus was set at 125 millicoulombs; the mean threshold was 100) was ensured to induce an acceptable convulsion duration between 15 seconds to 3 minutes. Thus in all sessions, ECT would have deemed ineffective if the convulsion had lasted less than 15 seconds and hence was not accounted as an effective ECT. In this state, the electric shock was repeated only once at the same session by 5–10% increase at repeated one. If convulsion had lasted longer than 3 minutes, patients would have been introduced to a neurologist for examination; however, this did not occur in this study. 6–12 ECT sessions were held for recruited patients, three times per week.

## 3. Measurements

### 3.1. Wechsler Memory Scale-Revised

Wechsler Memory Scale (WMS) was designed in 1970 by Wexler. The Revised WMS (WMS-R) consists of 5 subscales (general memory, attention/concentration, verbal memory, visual memory, and delayed recall) and evaluates different aspects of memory. Psychometric properties of Farsi version of WMS-R have been evaluated in Iran, in people aged 16 to 64 years. In this study, test-retest reliability coefficients were reported as 0.28 to 0.98 for the subscales and compositions. The average raw scores of the two groups of clinical and normal subjects were compared to evaluate the validity of WMS-R, indicating that the mean raw scores of the clinical group were significantly lower than that of the normal subjects [14].

### 3.2. Hamilton Rating Scale for Depression

The Hamilton Depression Rating Scale (HRSD) is a 21-item multiple-choice scale which assesses various dimensions of depression: behavioral, physical, emotional, guilt, hypochondriasis, sex issues, job, suicide, and sleep disorders. This scale was used in this study to assess the severity of depression and to match the groups. The reliability and validity of the scale have been confirmed in several studies [15].

### 3.3. Structured Clinical Interview for DSM-IV (SCID-IV)

This is a structured clinical interview based on DSM-IV criteria, for diagnosing MDD and other psychiatric disorders associated with Axis I and Axis II. SCID-IV has been used more than any other forms of psychiatric diagnostic interviews in psychological studies and has a global credibility. The reliability and validity of SCID-IV have been assessed in Iran, showing an acceptable reliability and validity [16].

### 3.4. Statistical Methods

All statistical analyses were performed using SPSS version 21. The data are reported by means of descriptive statistics (i.e., mean, SD, frequency, and percentage). Independent *t*-test was used to compare the significance of difference between the control and test groups. In addition, paired *t*-test was employed to determine differences in pretest and posttest mean scores from WMS-R in each group. Analysis of covariance (MANCOVA) was used to compare the difference between the posttest scores of both groups in WMS-R, with controlling the pretest. *P* values less than 0.05 were considered significant.

## 4. Findings

Sixty patients were enrolled in the liothyronine (*n* = 30) and placebo groups (*n* = 30). The minimum and maximum ages of the patients were 20 and 50 years. Most patients were female (76.7% of target group and 70% of controls), with an education level of diploma or higher (academic), married, housewife, and from urban area. All patients were diagnosed with major depressive disorder, without comorbidities or history of ECT in the recent six months. The majority of patients underwent 7 sessions of ECT. Demographic characteristics of these patients are presented in Table 1.

Table 2 depicts the scores of WMS-R in the liothyronine and placebo groups. A paired *t*-test was applied to scores of patients receiving placebo and no significant change was observed in verbal memory and general memory, while visual memory, attention/concentration, and delayed recall scores were significantly reduced after ECT (*P* < 0.05).

In patients receiving liothyronine, visual memory and attention/concentration had no significant change after ECT, but verbal memory and general memory scores were significantly increased in posttest measuring (*P* < 0.01).

The univariate analysis of covariance (with pretest control) was used to evaluate the effect of each group on all research's variables. Before using multivariate analysis of covariance (MANCOVA), normal distribution of the dependent variables and the associated variables, variance homogeneity and covariance homogeneity of the groups, and the weakness of correlation between the variables were ensured.

Table 3 shows that difference in the mean scores of verbal memory between the two groups in posttest is significant (*F*(1,25) = 13.95, MS = 1149.05, *P* = 0.001, and Eta^2^ = 0.21). Difference in the mean scores of visual memory between the two groups in posttest is significant in both groups (*F*(1,25) = 5.03, MS = 785.70, *P* = 0.02, and Eta^2^ = 0.09). Difference in the mean scores of general memory between the two groups in posttest is significant (*F*(1,25) = 13.45, MS = 1744.04, *P* = 0.001, and Eta^2^ = 0.20). Difference in the mean scores of attention/concentration between the two groups in posttest is significant (*F*(1,25) = 5.87, MS = 795.05, *P* = 0.05, and Eta^2^ = 0.10). Difference in the mean scores of delayed recall between the two groups in posttest is significant (*F*(1,25) = 19.27, MS = 1662.12, *P* = 0.001, and Eta^2^ = 0.27). Therefore, it can be concluded that liothyronine effectively increases memory functioning (verbal memory, visual memory, general memory, attention/concentration, and delayed recall). Based on Eta square index, 9%–27% of improvement in verbal memory, visual memory, general memory, attention/concentration, and delayed recall was due to liothyronine.

## 5. Discussion

The results of this study showed that liothyronine can prevent memory deficit after ECT in patients with MDD.

Despite clinical indication, patients may disagree to start or discontinue the ongoing treatment course with ECT [17] because of its well-known cognitive side effects [18]. Several efforts have been made to find a practical and effective preventive treatment for memory deficits after ECT. Add-on strategies seem to be the most popular [19]. Promising outcome has been reported by previous studies using thyroid hormone as an add-on strategy to ECT. These studies, not many, have not influenced guidelines yet, because of diversity in methodology and their limitations.

The current study matches up with previous reports in terms of effect of liothyronine. Stern et al. [20] randomized 20 patients with MDD and reported that patients receiving liothyronine (*n* = 11) performed better in measure of remote personal memory, but not learning or recall. Hamidia et al. [21] also reported that liothyronine could prevent memory deficits after ECT in patients with MDD. Masoudzadeh et al. [22] stated that patients who received liothyronine achieved better scores on the depression and memory scales, beginning after 6 sessions of ECT. Both of latter studies also had a small sample size (40 and 30 in that order) but showed improvement on both the Hamilton Rating Scale for Depression and the Revised Wechsler Memory Scale.

Stern et al. [23] repeated the same trial with 30 patients later and liothyronine could improve verbal learning performance and remote memory. The present study replicated these results with a larger sample size.

Most of the previous studies did not examine memory subscales. Our findings show that the mean scores of the experimental group in terms of verbal memory and general memory had an improvement attributable to the use of liothyronine, though this improvement was small.

Different mechanisms have been proposed to explain the discussed effect of liothyronine. A neuroprotective effect has been confirmed for triiodothyronine (T3) when electroconvulsive shock is administered to rats [24]. Additionally, anticonvulsive effect of thyrotropin releasing hormone will diminish when it is suppressed by exogene liothyronine. This may lower the required dose of electroshock and consequently decrease the damage and adverse cognitive effects [22]. Additionally, Siegrist and Kaiser [25] believe that the number of ECT sessions has no impact on memory and that decreased thyroxine (T4) may be the reason for the reduced rate of neural actin polymerization. Thus, when the level of T3 declines in patients receiving liothyronine, this leads to a temporary disruption in actin cytoskeleton and could protect cells from destructive effects of convulsion in amygdala and hippocampus neurons. These two constructs are very sensitive to convulsion and play an important role in memory and learning.

This study had some limitations. In this study, the interactive effects of individual variables and variables such as the severity of depression and thyroid hormone alterations were not studied. We also suggest to conduct a study on patients with MDD who receive liothyronine for preventing cognitive effects of ECT but still suffer from symptoms of memory deficit after ECT to find predictive characteristics of effectiveness of such treatment. Selecting patients with MDD provided a homogenous sample; however, other studies with different samples are required to ensure that the beneficial effect is not only limited to depressed patients. We were also unable to take the seizure threshold into account or the stimulus charges of the effective electric shock.

## 6. Conclusion

The results showed that liothyronine improves the performance of verbal memory, visual memory, attention/concentration, and delayed recall in patients with MDD who receive ECT. This study, adds to the evidence that liothyronine may have a positive impact in preventing memory impairment caused by ECT in depressed patients.

## Figures and Tables

**Table 1:** Demographic and individual characteristics of the patients undergoing ECT; each group included thirty patients.

	Liothyronine	Placebo
	*N* (%)	*N* (%)
Age	34.90 ± 8.06^*^	33.60 ± 7.99^*^
Hamilton depression score	62.56 ± 5.82^*^	62.63 ± 6.96^*^
Gender		
Male	7 (23.3)	9 (30)
Female	23 (76.7)	21 (70)
Education level		
Primary school	7 (23.3)	8 (26.7)
Middle school	7 (23.3)	8 (26.7)
Graduate/postgraduate	16 (53.4)	14 (46.6)
Marital status		
Single	9 (30)	7 (23.3)
Married	20 (66.7)	17 (56.7)
Divorced/widowed	1 (3.3)	6 (20)
Occupation		
Employee	9 (30)	12 (40)
Housewife	21 (70)	18 (60)
Location		
City	24 (80)	24 (80)
Village	6 (20)	6 (20)
Number of ECT sessions		
Six	6 (20)	4 (13.3)
Seven	14 (46.7)	19 (63.9)
Eight	1 (3.3)	0
Ten	4 (13.3)	5 (16.7)
Twelve	5 (16.7)	2 (6.7)

^*^As mean ± standard deviation.

**Table 2:** Mean and standard deviation of the WMS-R and the results of paired *t*-test in both groups.

Variables	Liothyronine			Placebo		
	Pretest	Posttest	*P* value	Pretest	Posttest	*P* value
Verbal memory	67.53	75.63	0.001	65.43	63.83	0.15
Visual memory	80.70	81.66	0.74	73.10	67.36	0.005
General memory	68.46	74.20	0.005	61.73	57.76	0.21
Attention/concentration	92.06	94.90	0.33	86.73	82.56	0.01
Delayed recall	72.03	76.06	0.066	64.43	60.40	0.02

**Table 3:** Results of univariate analysis of covariance of liothyronine effect on memory posttest with pretest control.

Variables	Sum of squares	df	Mean square	*F*	*P* value	Eta^2^	Power
Verbal memory	1149.05	1	1149.05	13.95	<0.001	0.21	0.95
Visual memory	785.70	1	785.70	5.03	0.025	0.09	0.61
General memory	1744.04	1	1744.04	13.45	0.001	0.20	0.94
Attention concentration	795.05	1	795.05	5.87	0.019	0.10	0.66
Delayed recall	1662.12	1	1662.12	19.27	<0.001	0.27	0.99
